# The Use of Volatile Anesthetics as Sedatives for Acute Respiratory Distress Syndrome

**DOI:** 10.31480/2330-4871/084

**Published:** 2019-02-21

**Authors:** Sophia Koutsogiannaki, Motomu Shimaoka, Koichi Yuki

**Affiliations:** 1 Department of Anaesthesia, Harvard Medical School, Boston, Massachusetts, USA; 2 Department of Molecular Pathobiology and Cell Adhesion Biology, Mie University Graduate School of Medicine, Tsushi, Mie, Japan; 3 Department of Anesthesiology, Critical Care and Pain Medicine, Cardiac Anesthesia Division, Boston Children’s Hospital, Boston, Massachusetts, USA

## Abstract

Acute respiratory distress syndrome (ARDS) remains to pose a high morbidity and mortality without any targeted therapies. Sedation, usually given intravenously, is an important part of clinical practice in intensive care unit (ICU), and the effect of sedatives on patients’ outcomes has been studied intensively. Although volatile anesthetics are not routine sedatives in ICU, preclinical and clinical studies suggested their potential benefit in pulmonary pathophysiology. This review will summarize the current knowledge of ARDS and the role of volatile anesthetic sedation in this setting from both clinical and mechanistic standpoints. In addition, we will review the infrastructure to use volatile anesthetics.

## Current Status of Acute Respiratory Distress Syndrome

The respiratory-distress syndrome of tachypnea, refractory hypoxemia, and diffuse opacities on Chest X-ray was first described in 1967 [1]. This was later called acute respiratory distress syndrome (ARDS), and its diagnosis criteria was defined in 1994 by the North American European Consensus Conference (NAECC), as 1) Acute and sudden onset of severe respiratory distress, 2)Bilateral infiltrates on Chest X-ray, 3) The absence of left atrial hypertension, and 4) Severe hypoxemia (PaO_2_/ FiO_2_ <= 200 mmHg) [2]. Flooding of the distal airspaces with protein-rich edema fluid is largely responsible for hypoxemia [3]. The term “Acute lung injury (ALI)” was defined as an entity that meets 1) - 3) above and has less severe hypoxemia (PaO_2_/FiO_2_ <= 300 mmHg). However, a number of issues were raised regarding the NAECC definition. The ARDS Definition Task Force redefined ARDS in 2012 (as follows) and the term ‘ALI’ was eliminated; 1) Onset within 7 days after a known clinical insult or new or worsening respiratory symptoms, 2) Bilateral opacities on chest radiograph, and 3) Hypoxemia (PaO_2_/FiO_2_ <= 300 mmHg) in the presence of a minimum positive end-expiratory pressure (PEEP) of 5 cm H_2_O (‘Berlin definition’) [4]. Left atrial hypertension was no longer included because the usage of pulmonary artery catheters had been declining and ARDS could co-exist with high left atrial pressure. However, it was clearly stated that hydrostastic edema could not be the primary cause of ARDS. If risk factors were not identified for ARDS, this new definition mandated to exclude hydrostatic edema as a cause of respiratory failure. The risk factors for ARDS are listed in [5,6]. Among them, pneumonia (59.4%), extrapulmonary sepsis (16.0%) and aspiration (14.2%) were the major risk factors of ARDS in the recent study [7]. ARDS was categorized based on the degree of hypoxemia as follows; mild - PaO_2_/FiO_2_ 200–300 mmHg, moderate- PaO_2_/FiO_2_ 101–200 mmHg, and severe - PaO_2_/FiO_2_ <= 100 mmHg.

In an international study involving 50 countries, ARDS, diagnosed using the Berlin definition, was observed in 10% of all the patients who admitted to ICU and in 23% of mechanically ventilated patients [7]. The estimated annual incidence of ARDS using data from 1999 to 2000 was 190,600 cases in the U.S. (Of note, in this study, onset criteria and PEEP requirement mandated in the Berlin definition was not used for ARDS diagnosis) [8]. The mortality of patients with severe ARDS was extremely high (46%) in the aforementioned international study [7]. This result was consistent with the mortality of Berlin definition validation cohort (mortality of mild, moderate and severe ARDS was 27%, 32% and 45%, respectively) [4]. Many of patients with ARDS also develop non-pulmonary organ failure [6]. Survivors may suffer from neuromuscular dysfunction (neuropathy, myopathy), neurocognitive dysfunction (abnormality in memory, attention, concentration), and neuropsychological dysfunction (depression, anxiety), which could leave long-term consequences [8]. Thus, reducing the incidence and attenuating the disease progression is warranted [9].

However, currently there is no specific therapy against ARDS. The mainstay of ARDS management is to identify and treat the underlying causes of ARDS. For example, treatment for pneumonia should be the priority if this is an inciting disease. For ARDS itself, supportive management is used to limit further lung injury. Supportive management associated with the improvement of ARDS outcome includes limiting of tidal volume and plateau pressure, use of neuromuscular blockade, use of prone position and conservative fluid administration [10–13]. Some of the groundbreaking work are introduced here; In a groundbreaking trial comparing low-tidal volume (6 mL/Kg) versus high tidal volume (12 mL/Kg) ventilation testing all the severity of ARDS patients, the mortality during the first 180 days was 31.0% in the low tidal volume group and 39.8% in the high tidal volume group [10]. Using conservative fluid administration over liberal fluid administration to this population shortened the duration of mechanical ventilation, but did not show survival benefit [13]. Prone position and neuromuscular blockade was tested in moderate-to-severe ARDS (PaO_2_/FiO_2_ < 150 mmHg). Patients with only deep sedation group (control group) were compared with patients with deep sedation who received cis-atracurium for 48 hours (muscle relaxant group) [12]. The 28-day mortality was 23.7% in the muscle relaxant group and 33.3% in the control group, and the 90-day mortality was 31.6% and 40.7%, respectively. The 28-day mortality was 16.0% in the prone group and 32.8% in the supine group, and the 90-day mortality was 23.6% in the prone group and 41.0% in the supine group [11]. The American Thoracic Society, European Society of Intensive Care Medicine and Society of Critical Care Medicine proposed clinical practice guideline for mechanical ventilation based on a number of clinical trials [14]. In addition, sedation regimen and neuromuscular blockade have been reviewed and their clinical guideline was suggested [15–17]. Current recommendation for ARDS management is summarized.

A number of pharmacological interventions for ARDS have been attempted without success [18]. While the development of specific pharmacological therapy is necessary and continues to be explored, a body of research has suggested that sedative choice, particularly use of non-authentic sedative volatile anesthetics could benefit the outcome of ARDS [19–23]. Here we will review the current knowledge of sedatives in ARDS and the role of volatile anesthetics.

## Volatile Anesthetics as Sedatives in Patients with ARDS

### The goal of sedation and its role in the outcome

In patients with ARDS, sedation is used to improve tolerance of mechanical ventilation, reduce discomfort, and improve patient-ventilator synchrony [16]. Inadequate sedation can cause agitation, accidental extubation, or hemodynamic instability. With the introduction of electronic flow triggering [24], synchronization became a less important indication. Because of adverse effects on clinical outcomes posed by stress and anxiety [25], judicious sedation was often provided to mitigate exposure to psychological disturbance [26].

As a result, over-sedation was commonly observed (40–60% of patients) [16,27,28]. The contribution of over-sedation to adverse outcomes was pointed out by a number of studies [29–32]. The depth of sedation was independently associated with the duration of mechanical ventilation (MV), in-hospital mortality, and rate of death [27,31,33,34]. Surprisingly, lighter sedation was not associated with psychological adverse outcomes [35–37]. In addition, delirium was less frequent under lighter sedation [16]. Although not all, a significant portion of patients examined in these studies had ARDS [29–32], suggesting that these results were relevant to patients with ARDS [16]. The 2018 Pain, Agitation/sedation, Delirium, Immobility (rehabilitation/mobilization), and Sleep (disruption) (PADIS) guideline recommends light sedation over deep sedation for ICU patients [38]. Although patients with severe ARDS are often ventilated with low tidal volume and high PEEP, deep sedation is not necessarily required for this purpose [39–43]. However, deep sedation is required for patients on neuromuscular blockade, and possibly for prone position and ECMO use [44–46].

The majority of sedatives and analgesics are given intravenously [47]. Midazolam, lorazepam, diazepam, dexmedetomidine, ketamine, remifentanil, fentanyl, morphine and hydromorphone are the mainstay for sedation. Benzodiazepines and propofol are used in 60% and 20% of cases, respectively [27]. Because sedatives are often given continuously, the context-sensitive half-time (CSHT) rather than the terminal elimination half- lifeis proposed as a more clinically relevant measure[48]. The CSHT describes the time required for the plasma drug concentration to decline by 50% after terminating an infusion. It depends on both distribution and metabolism of a given drug,and predicts recovery from infusion more accurately [49]. Decreased hepatic and renal blood flow leads to change in metabolism and clearance [50]. CSHT usually increases as the duration of infusion goes longer. Midazolam, lorazepam, diazepam, propofol, ketamine, fentanyl, morphine and hydromorphone, for example, can have longer CSHTs due to slow metabolism and clearance in critically ill patients. Remifentanil, metabolized by plasma and tissue esterases has an extremely short CSHT (2.45 min after 3-hour infusion [48]), but can cause acute development of withdrawal and tolerance [51]. Dexmedetomidine with CSHT of one hour [52] is increasingly in use. Protocol-directed sedation protocol, daily interruption of continuous sedation, and spontaneous breathing trial have been used with good effect and recommended in the PADIS guideline [29,31,38,53]. Validated sedation scales and protocols should be used to titrate sedation [49].

The PADIS guideline also described preference of propofol or dexmedetomidine over benzodiazepines [38]. Benzodiazepine was associated with an increased mortality over propofol or dexmedetomidine [54].

The ideal sedative should have a rapid onset and offset of action, and allow precise titration of sedation without accumulation after long-term use [55]. However, currently intravenous sedatives do not meet these criteria perfectly. As alternatives, volatile anesthetics (VAs) have been introduced as sedatives in ICU in Europe and Canada [56], and some countries list them as alternative sedatives in the sedation guideline [57]. Currently they are not in a part of the PADIS guideline. Isoflurane, sevoflurane and desflurane are commonly used VAs. They are promiscuous, small molecules that interact with several receptors in the central nervous system such as GABA_a_ receptor, N-methyl-D-asparate (NMDA) receptor and tandem pore domain potassium channel (K2P). Their CSHTs are comparable and do not increase with the duration of administration (CSHT of < 10 min) [58]. In the meta-analysis, VA sedation did not increase short-term adverse events, and was associated with a reduction in time to extubation [59]. The majority of reports are based on short-term use, and the assessment of long-term use is in progress.

### Benefits of volatile anesthetics in ARDS settings

As mentioned above, VAs have favorable CSHT profile. So far there is no study examining the effect of VAs on delirium in ICU setting. Isoflurane, sevoflurane and desflurane demonstrated a trend in the reduction of post extubation agitation, delusion, negative feelings and factual ICU memory over midazolam or propofol sedation in some studies [60–62].

VAs may have favorable features on non-sedative aspects including lung pathology. The retrospective study by Bellgardt, et al. examined the mortality of patients on ventilator under isoflurane or propofol/ midazolam [63]. Isoflurane arm (0.3–0.8%) had a significantly lower mortality than propofol/midazolam arm. Isoflurane arm also had shorter ventilator-support, in line with other studies that VA group experienced earlier extubation (sevoflurane 0.5–1.0%, isoflurane 0.1–0.6%) [60,64–66]. Early extubation may potentially reduce ventilator-associated complications such as atelectasis, volutrauma and pneumonia. The effect of sedation onpulmonary function such as gas exchange was not examined in this study. The study by Jabaudon, et al. suggested that VA might offer direct benefit to pulmonary function. They prospectively compared PaO_2_/FiO_2_ of ARDS patients who received sevoflurane (mean 0.6–0.7%) or midazolam sedation for 48 hours [22], and found that sevoflurane arm showed higher PaO_2_/FiO_2_.

With the limited number of studies available in ICU settings, the studies in operating room settings can present additional insight. In the meta-analysis by Uhlig, et al., general anesthesia with VAs was associated with reduced mortality and lower incidence of pulmonary complications over intravenous anesthetics (lAs) after cardiac surgery [67]. The outcome did not differ between the two groups undergoing non-cardiac surgery, but this may be due to significant heterogeneity in cases enrolled. In the prospective study by Grabitz, et al., higher VA doses were associated with less pulmonary complications, lower 30-day mortality and lower cost in non-cardiac surgeries [68]. Higher doses were beneficial only in patients without prolonged intraoperative hypotension, suggesting tissue injury via impaired perfusion needs to be avoided. In the prospective study by De Conno, et al. sevoflurane anesthesia showed lower pro-inflammatory mediator levels along with less postoperative (mostly lung related) complications than propofol anesthesia in surgery requiring one- lung ventilation [69]. One-lung ventilation and use of hyperoxia involves a number of physiological changes, and the data need to be interpreted with caution. The effect of different VAs and doses should be examined in diverse patient population in the future. Additional feature of VAs is that it can induce muscle relaxation. In severe ARDS, muscle relaxation can be used as mentioned above. Thus, the property of muscle relaxation by VAs potentially work in favor.

### Mechanism of volatile anesthetics-induced modulation of ARDS

The findings that VAs might work favorably for lung pathophysiology including ARDS are exciting, but it is important to understand the underlying mechanism. At the alveolar level, oxygen and carbon dioxide need to diffuse efficiently across the alveolar-capillary membrane. As the lung as a whole, alveolar ventilation (V) and pulmonary circulation (Q) needs to be matched. In healthy volunteers, VAs worsen V/Q matching [70], which does not explain the aforementioned favorable pulmonary effects. Of note, similar study has not been done using IAs or patients with lung injury. The carbon monoxide diffusion capacity (DLco) is the most sensitive measurement of alveolar-capillary gas transfer [71]. This has not been tested in human subjects under different sedatives. Its measurement in rodents is possible [72], but has not been done under different sedatives. In general, the mechanism was limitedly analyzed in clinical studies. Preclinical studies are insightful to address the mechanism of lung injury and the effect of different sedatives. Thus, we will go over the molecular mechanism of ARDS and the proposed mechanism of VA-induced ARDS modulation illustrated in preclinical studies in the followings.

### Lung injury in ARDS

ARDS can be categorized into three phases (acute, subacute, and chronic) [73]. In the acute phase, interstitial and alveolar edema with accumulation of neutrophils, macrophages, and red blood cells in the alveoli is seen. Often denuded alveolar epitheliums and hyaline membranes are observed. As a result of tissue injury, lung develops significant permeability. Non-cardiogenic pulmonary edema is a signature of ARDS, and develops because of an increase in fluid influx from the vasculature into the alveolar airspaces, and a reduction in normal capacity of the alveolar epithelium to remove edema fluid from the airspaces (alveolar fluid clearance) [3,74]. In the subacute phase, some of the edema is reabsorbed with sign of repair including proliferation of alveolar epithelial type (AT) II cells. In the chronic phase, there is a resolution of the acute neutrophilic infiltrate and fibrosis with ongoing evidence of alveolar epithelial repair.

**Figure 1:**
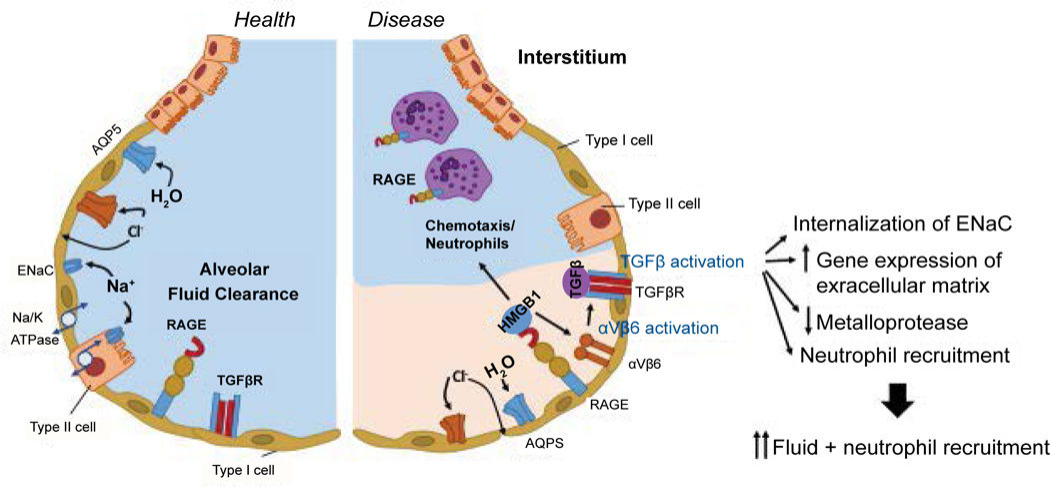
The scheme of alveolus in healthy and injured lung. In healthy lung, alveolar fluid clearance occurs normally with the help of Na/K ATPase, Na channel (ENaC) and aquaporin (AQP5). TGF-β is not activated yet. In injured lung, ENaC is internalized and AQP5 expression is reduced, resulting in the impairment of alveolar fluid clearance. Integrin αvβ6 activates TGF-β. Also HMGB1 is released from dying cells and binds to RAGE on the alveolar epithelial cells. Neutrophils also have RAGE on their surface, and HMGB1 acts as a neutrophil chemoattractant.

Activated neutrophils release neutrophil elastase (NE). NE is a serine proteinase stored in azurophilic granules, and cleaves key endothelial cell-associated adhesion molecules to cause lung damage [75], Neutrophil extracellular traps (NETs) are net-like chromatin fibers decorated with neutrophil-derived components such as histones, myeloperoxidase (MPO) and NE. Histones and MPO are also cytotoxic to epithelial and endothelial cells. The involvement of NETs in lung injury has been shown [76]. The increased permeability of the alveolar-capillary barrier [76] and the impaired fluid clearance are responsible for early lung injury as described above. Fluid clearance is controlled by epithelial Na^+^ and Cl^−^ ion transport (Figure 1). Na^+^ transport is largely undertaken by the Na^+^/K^+^-ATPase and the epithelial sodium channel (ENaC). Increased transforming growth factor (TG- F)-β levels are observed in lung fluids from patients with ALI/ARDS [77,78]. Alveolar epithelial-restricted integrin avP6 activates TGF-β, stored at high concentrations in the extracellular matrix [79]. TGF-β1 acts as a neutrophil chemoattractant, and increases neutrophil respiratory burst, phagocytosis and survival [80]. It also facilitates internalization of ENac, leading to alveolar flooding [74,81]. TGF-β also directly increases the permeability of pulmonary endothelial monolayers and alveolar epithelial monolayers [81]. TGF-β also induces the genes expressing the extracellular matrix and inhibits metal-loprotease to seal off inflammation and facilitate tissue repair. The receptor for advanced glycation end-products (RAGE) is a membrane receptor in AT-1 epithelial cells [82]. RAGE is highly expressed in lung, and plays a significant role in pulmonary homeostasis, particularly cell spreading and growth. AT-1 cells occupy 95% of the lung epithelial cells, while AT-2 cells occupy 5%. RAGE is a pro-inflammatory molecule and increases its expression in inflammation. Soluble RAGE, produced by alternative splicing or truncation of membrane RAGE, acts as a decoy to bind to its ligands and attenuate further inflammation [83]. High-mobility group box 1 (HMGB1) is a non-histone chromatin-associated protein actively secreted or passively released from necrotic or injured cells, and serves as a ligand for RAGE [84]. HMGB-1- RAGE axis activates TGF-β via integrin αvβ6. RAGE is also expressed on neutrophils, and HMGB1 recruits neutrophils to the site of necrosis [85].

MV is an indispensable component of advanced life support, but it can damage the lung (ventilator-induced lung injury; VILI). VILI is caused by overdistension at high lung volumes (volutrauma), collapse/reopening of airway units at low lung volumes (atelectrauma) and activation of immune system (biotrauma) [86]. Volutrauma and atelectrauma represent mechanical trauma. Atelectrauma causes perforation in the airspaces and volutrauma enhance it [87], because atelectatic lesion poses lung at an increased risk of local strain for inflation [88]. Cyclic stretch of lung induces the inflammatory reaction and can affect systemic circulation and distal end-organs [89]. Cytokine production, neutrophil activation and subsequent tissue injury constitute biotrauma [90]. Neutrophil depletion attenuated VILI in rabbits [91]. Blocking interleukin (IL)-1 led to inhibition of neutrophil recruitment and less lung injury [92]. Neutrophils can cause VILI via NETosis [93] and release of NE [94]. The involvement of HMGB-1-RAGE [95,96] and TGF-β [97] in VILI has been described as above.

### The mechanism of volatile anesthetics-induced reduction in lung injury

A growing evidence indicates the immunomodulatory effects of VAs [98,99]. The role of VAs in lung pathophysiology was tested mostly in lipopolysaccharide (LPS)-induced lung injury models. Exposure of isoflurane before and after LPS instillation reduced neutrophil recruitmentand lung injury [100,101]. A number of pre-clinical studies identified neutrophils as central, cellular mediators of the early, innate immune response, causing damage to the lung [102]. Abundant accumulation of neutrophils has been seen in lung in patients with ARDS [103]. Thus, the modulation of neutrophil function by isoflurane could play a role in lung injury reduction. Similarly, sevoflurane exposure was associated with less lung injury and better oxygenation than propofol [104]. The effect of VAs on neutrophil function including neutrophil recruitment has been described *in vivo.* In the study of sepsis model, isoflurane attenuated neutrophil recruitment but propofol did not [105]. Neutrophils are recruited to organs and tissues via chemoattractants and adhesion molecules. Isoflurane and sevoflurane directly inhibit the function of adhesion molecules [105–107]. In addition, VAs can reduce proinflammatory levels. Sevoflurane exposure attenuated production of proinflammatory mediators in bronchoalveolar lavage (BAL) fluid [108]. This is in line with the study of patients with one-lung ventilation that VAs reduced alveolar inflammatory response, but propofol did not [109].

In addition to the effect of VAs on neutrophils, they affect alveolar epithelial cells. Isoflurane attenuated proinflammatory response by alveolar epithelial cells via atypical type A γ-aminobutyric acid receptors (GAB- A_a_ receptors) [110]. Similarly, halothane and enflurane reduced proinflammatory response [111]. Sevoflurane also attenuated proinflamatory response and attenuated apoptosis of epithelial cells [112]. Sevoflurane might enhance the function of ENaC and Na^+^/K^+^-ATPase on epithelial cells to mitigate pulmonary edema [23]. The benefit of VAs in lung injury was confirmed in another model. In post-hemorrhagic shock model, lung injury was attenuated by isoflurane over pentobarbital [21].

Isoflurane and sevoflurane also worked beneficially during MV. In primary VILI model, sevoflurane and isoflurane attenuated neutrophil recruitment, activation and VILI more over ketamine and desflurane anesthesia [113]. In another study, sevoflurane exposure during MV was associated with less oxidative burst and lower proinflammatory mediator levels in BAL [114]. Desflurane may not be as potent as isoflurane and sevoflurane, but further investigation is warranted to conclude. Isoflurane exposure attenuated VILI by inhibiting phos-phoinositide 3-kinase (PI3K)/Akt signaling [19]. The inhibition of PI3K/Akt signal exacerbates lung alveolar permeability and inflammation [114]. In the two hit model of LPS induced lung injury followed by MV, isoflurane and desflurane exposure maintained the integrity of the alveolar-capillary barrier [18]. So far the effect of VAs on RAGE and TGF-β has not been reported. We should also keep in mind that the preclinical studies were largely performed using sterile inflammation model [104]. A growing literature suggests that VAs pose immunomodulatory effects [97,98]. In fact, prolonged exposure to isoflurane can cause neutrophil dysfunction, worsen bacterial loads and outcomes in the setting of sepsis [104]. Because patients with ARDS could have impaired immune function, this potential immunomodulatory effects by VAs should be kept in mind when VAs will be used for patients with sepsis for a long duration.

### Practical aspect of volatile anesthesia usage in ICU setting

**Figure 2:**
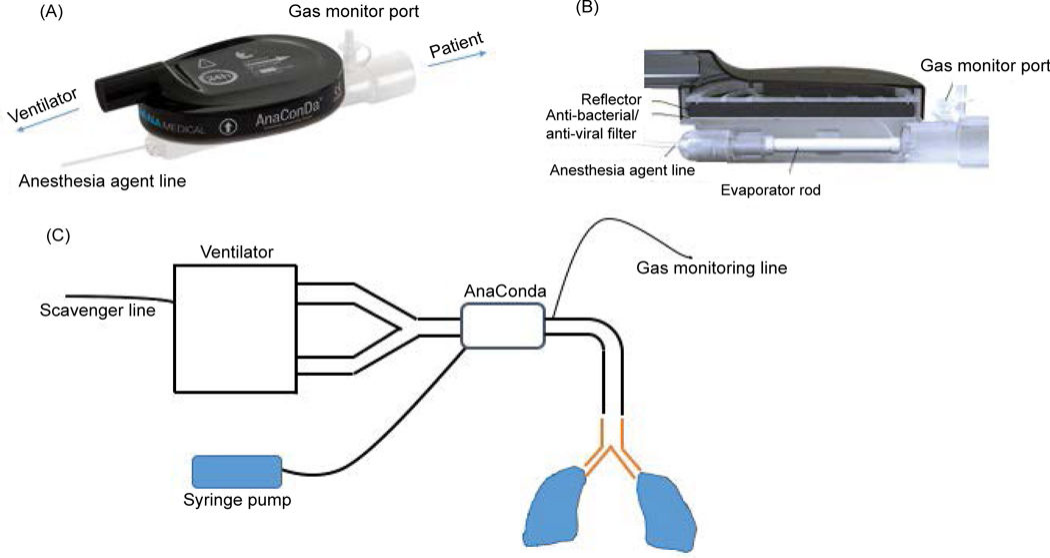
The AnaConDa device and its setup. (A, B) The design of the AnaConDa device. (C) The setup for the AnaConDa device use

In general, VAs at one-third of doses for general anesthesia would be adequate to achieve sedation [116]. This is illustrated in the studies cited above [22,60,63–66]. However, VAs at much higher concentrations are required when deeper sedation is indicated [116]. VAs are mainstay drugs for general anesthesia in the operating rooms and administered via vaporizers mounted on anesthesia machines with circular circuits. Because ICU ventilators uses high-flow, non-rebreathing, non-circular circuits, the vaporizers mounted on anesthesia machine are not adequate for use. The development of miniature vaporizers such as the Anesthesia Conserving Device (AnaConDa) [117] or MIRUS system simplified the use of VAs on ICU ventilators (Figure 2A and Figure 2B) [118]. A couple of technical issues should be noted. The AnaConDa or MIRUS system is typically placed between the Y-piece and the patient (Figure 2C). AnaConDa can accommodate isoflurane, sevoflurane, but not desflurane. The large dead space (100 mL) limits its pediatric use. Some advocate placing this in the inspiratory limb to use in children with cost of no recycling of VAs. 90% of VAs are absorbed on the activated carbon fibers during expiration and recycled back to patients, but 10% of the vaporized gas require scavenging by incorporating an active or passive scavenging system to the expiratory outlet of the ventilator [116]. For passive gas adsorption, charcoal canisters are used. For active gas adsorption, waste gases are siphoned to the main hospital waste gas outlet system. The association of high atmospheric VA levels with infertility and spontaneous abortions led to the recommendation that occupational atmospheric levels should be maintained below less than 2 parts per million (ppm) in North America [116]. Monitoring VA concentration in ICU environment using infrared spectroscopy should be performed to ensure that VA level in ICU is below the recommended range. The MIRUS system is compatible with desflurane [119,120]. Both are not available in the US. VAs have been given patients with status asthmaticus and status epilepticus by anesthesia machine in ICU in the US [116,121,122].

The potential problem of VAs should be noted. Malignant hyperthermia can be triggered with the use of VAs. One case has been reported in ICU use [123]. This is quite rare with the incidence of 1: 5,000–1:50,000–100,000 [124,125]. In contrast, propofol infusion can be more frequently seen (about 1:100) [126]. In addition, environmental aspect needs to be considered. The effect of VAs on global warming potentials has been reported. Desflurane accounts for the largest life cycle greenhouse gas emissions among all the VAs with 15 times that of isoflurane and 20 times that of sevoflurane [127]. Due to this concern and the potential weaker lung protective property shown in a preclinical study, desflurane may not be the priority drug for ICU use in patients with ARDS. Lastly, VA administration is currently only trained during anesthesia training. Thus, the presence and/or immediate availability of a board certified anesthesiologist should be also taken into consideration when VAs are needed to administered to a patient for sedation.

## Future Direction

Although VAs showed favorable profiles in preclinical and clinical studies, larger clinical studies need to be performed to potentially facilitate VA-based sedation in ICU setting to determine its safety and benefit. Preclinical studies should also supplement further knowledge.
